# Marketing and clinical trials: a case study

**DOI:** 10.1186/1745-6215-8-37

**Published:** 2007-11-20

**Authors:** David Francis, Ian Roberts, Diana R Elbourne, Haleema Shakur, Rosemary C Knight, Jo Garcia, Claire Snowdon, Vikki A Entwistle, Alison M McDonald, Adrian M Grant, Marion K Campbell

**Affiliations:** 1 Centre for Research and Innovation Management, Brighton, UK; 2 Nutrition and Public Health Interventions Research Unit, London School of Hygiene and Tropical Medicine, Keppel Street, London, UK; 3 Medical Statistics Unit, London School of Hygiene and Tropical Medicine, Keppel Street, London, UK; 4 Centre for Family Research, University of Cambridge, Free School Lane, Cambridge, CB2 3RF, UK; 5 Health Services Research Unit, University of Aberdeen, Health Sciences Building, Foresterhill, Aberdeen, UK

## Abstract

**Background:**

Publicly funded clinical trials require a substantial commitment of time and money. To ensure that sufficient numbers of patients are recruited it is essential that they address important questions in a rigorous manner and are managed well, adopting effective marketing strategies.

**Methods:**

Using methods of analysis drawn from management studies, this paper presents a structured assessment framework or reference model, derived from a case analysis of the MRC's CRASH trial, of 12 factors that may affect the success of the marketing and sales activities associated with clinical trials.

**Results:**

The case study demonstrates that trials need various categories of people to buy in – hence, to be successful, trialists must embrace marketing strategies to some extent.

**Conclusion:**

The performance of future clinical trials could be enhanced if trialists routinely considered these factors.

## Background

Results from randomised controlled trials (RCTs) make an important contribution to improving patient care. Some trials recruit a large number of patients and involve the collaboration of many doctors, nurses and other healthcare workers around the world. Because trials (especially large trials) can involve a substantial commitment of time and money, it is essential that they address important questions and use rigorous scientific methods. More recently, however, it has been recognised that good management and effective marketing are also essential to enable sufficient numbers of participating centres and patients to be recruited so that the study has enough statistical power [1]. This paper reports a case study of a novel application of a marketing approach from the world of business to a single clinical trial in order to develop a reference model for use in other trials.

### Orientating concepts

Businesses strive to find customers and encourage them to buy what is on offer. Clinical trials strive to find doctors and patients and encourage them to sign up. Thus they face similar challenges and may need to adopt similar approaches to achieve their goals.

Clinical trials progress through distinctive stages, including study design, obtaining funding, finding participants, collecting and processing data, interpreting the results, and reporting. In some stages of a trial the key requirement is to do good science. However, in others the challenge is quite different – the key requirement is to install and operate a range of effective management techniques, similar to those required for marketing a product. Indeed, an experienced trialist observed that a trial is one fifth structure (science) and four-fifths process (i.e. management).

Marketing – the process of finding, winning over and retaining customers – is an important topic in management studies. Marketing has distinctive frameworks, methods and techniques – generally drawn from sociology and social psychology. Marketing became better understood in the 1960s [2] and now the discipline is ubiquitous within larger companies and many not-for-profit organisations. A definition of marketing by McDonald and Wilson [3] describes it as "a process for defining markets, quantifying the needs of the customer groups (segments) within these markets, determining the value propositions to meet these needs, communicating these value propositions to all those people in the organisation responsible for delivering them and getting their buy-in to their role, playing an appropriate part in delivering these value propositions to the chosen market segments (and) monitoring the value actually delivered" (pp11). (A value proposition can be defined as a clear statement of the tangible results a customer gets from using the products or services).

The marketing dimension is included only tangentially in the literature on clinical trials [1]. For example, trials are generally stated to need recruitment strategies, use of media and data tracking systems. However, the notion of a developing and working to achieve a formal *marketing plan *that covers all of the areas in the McDonald and Wilson definition is generally absent from descriptions of trial management.

This is not to suggest that trial managers consider the topic of marketing their trials to potential recruits lightly. Indeed, it is a dominant concern for many trialists. For example, the Diabetic Retinopathy Awareness Program study [4] undertook many initiatives to recruit volunteers and concluded, "these experiences substantiate the need for a comprehensive coordinated approach, using planned sources, to achieve recruitment success" (pp432). Farrell [5] has argued persuasively that it is lack of solutions to managerial issues that reduce the effectiveness of trials, and Rowe [6] suggested that, "to get patients into trials more efficiently pharma companies must begin to think like marketers".

It can be argued that marketing is *especially *important in clinical trials. Participation in a trial is a *formal *voluntary act, in that participants need to abide by a set of rules. Accordingly, not only is it necessary for people to volunteer, they also need to sign-up to behave in accordance with a set procedure [7]. In short, participants in a trial (be they clinicians or patients or their families) need to make a commitment, and undertake additional work, often without direct financial benefit to themselves.

**Figure 1:**
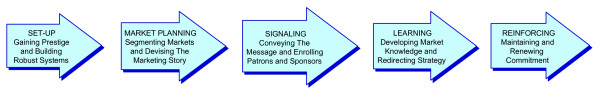
Five stages in marketing a trial.

From a marketing perspective, conducting a successful trial can be seen as a process with five main stages (Figure 1). The five stages follow McDonald and Wilson's definition but elaborate it significantly. The purposes and content of each stage is amplified in Table 1.

**Table 1:** Activities within the five stages in marketing a trial

Stage	Marketing Purposes
Set-Up	1. To gain the buy-in of the necessary authorities and stakeholders. 2. To gain the buy-in of opinion leaders whose explicit approval provides legitimacy and prestige for the trial. 3. To construct a marketing function within the trial and devise robust systems for ensuring that the marketing (and later sales) activities are undertaken efficiently, effectively and in accordance with the values and goals of the trial.

Market Planning	1. To identify and describe the distinctive features of the 'segments' of the 'market' to be targeted. 2. To discover what people in each of the selected market segments value (i.e. what would encourage them to 'sign-up'). 3. To develop a 'value proposition' (or more than one if required) that can be tested with each of the targeted segments. 4. To enrol the whole trial organisation in working within the trial's 'marketing brief'.

Signalling	1. To convey, fully and persuasively, the 'value proposition' to sufficient numbers of people in the target market. 2. To convey, fully and persuasively, the 'value proposition' to intermediaries (e.g. doctors or nurses), influencing bodies (e.g. ethics committees) and other agents that can either help or hinder the conduct of the trial.

Learning	1. To learn, through doing, about 'the market'. 2. To utilise ongoing learning to develop more effective policies and practices. 3. To evaluate, and redirect the strategy of a trial as learning is acquired.

Reinforcing	1. To maintain momentum by renewing or upgrading 'the offer' made to participants. 2. To sustain commitment of interested parties and other agencies whose support will be needed.

Clinical trials require strategy, management, marketing and sales. Undoubtedly they undertake the activities listed in the table 1 in some way. However, what happens if those who define the strategy of a trial, establish its management processes, devise its marketing plan and attempt to sell the benefits of participation try to improve their practice by explicitly engaging with the discipline of management? The study described below provides some preliminary answers to this question.

## Methods

This study is a component of a three-part project – STEPS (Strategies for Trials Enrolment and Participation) [8]. The first part included a quantitative analysis of the association between different patterns of recruitment in trials and factors thought likely to influence this pattern, based on an examination of Medical Research Council (MRC) and Health Technology Assessment (HTA) records [9]. This showed recruitment often fails to meet targets. The second part explored these issues further using qualitative analysis of transcripts from semi-structured interviews with key players in four trials considered by MRC/HTA as exemplars [10], with a particular focus on the complexity of financial negotiations. Here we report the third part based on an in-depth investigation of a single trial from a business perspective to assess its marketing strategy, in order to develop a reference model to aid future trials.

The Corticosteroid Randomisation after Significant Head injury (CRASH) trial [11] was a large scale RCT of the effect of corticosteroids compared to placebo in improving important health outcomes [12]. The trial aimed to recruit 20,000 head injured patients from hospitals world-wide. As the trial participants were unconscious the marketing strategy needed to focus on staff at participating hospitals (and not on the patients).

CRASH had a marketing challenge since it needed to engage the interest and collaboration of hundreds of people internationally, including members of ethics committees, surgeons, doctors, nurses and administrators. During the recruitment phase, one of us (DLF), a marketing and strategy specialist from the academic business sector, was invited to examine the trial as if it were a business, to comment on its marketing strategy and to help the trial team to understand and put in place a marketing plan over a two-year period. He was given access to all trial documents (apart from confidential investigators' personal details and patient data). He visited three participating hospitals in England, observed training sessions and interviewed or facilitated group discussions with doctors, nurses and ancillary staff. He also conducted 11 interviews, and held numerous meetings with members of the trial management team.

The methodological approach used techniques drawn from adaptive theory [13], case analysis [14] and action research [13]. The researcher's interview notes were analysed using a grounded theory framework [15] and the emerging model was compared with data from studies in commercial enterprises. The N-Vivo qualitative analysis software program was used to structure data initially but manual analytic methods were used later as often the purpose was to highlight what participating agents were *not *saying – rather than what they were saying. A professor of management (independent of the study team) checked the interpretative framework against the raw data. Emerging results were presented to a peer group (the STEPS research team) and to the team members of the CRASH trial. A one-day marketing workshop using an action research approach was held with the trial team to provide insights into the extent to which concepts and practices from the business world [15] might have relevance to management of clinical trials. Early in 2004 an additional five-hour workshop was held with representatives from three trials (one of which was the CRASH trial) to gain further insight into the practical implications of the findings, providing a further opportunity to validate the researcher's theory building process.

As the approach reported in this paper was part of a strategy to try to improve recruitment into the CRASH trial, the STEPS investigators decided that separate ethics committee approval would not be required for this process as the CRASH trial had already received approval from the North London MREC.

## Results

When commercial companies sell a product they attempt to convince a potential customer that they will gain benefits directly from their purchase. In the CRASH the trial managers were seeking to gain a commitment to engage from clinical professionals who would make no material gain for themselves. Accordingly, the CRASH trial was selling an opportunity for clinical professionals to participate in improving future clinical practice – an activity that can be seen as being akin to a charitable endeavour [7]. A challenge for the CRASH trial was to promote the idea that if a clinician signed up to the trial then medicine itself would progress and the clinician would be fulfilling a professional obligation.

A previously unexplored dimension of the marketing challenge was found to be the difficulty of gaining an evidence-based understanding the reasons why participants (in this case hospitals) signed up and what motivated them to fulfil a commitment that had no sanctions for non-performance. An analysis of feedback from participating hospitals concluded that they opted in for a variety of reasons, including the perceived merits of the study, the stature of the sponsors and advocates, the status provided to participants through participation and the affordability of participation (i.e how much time and effort would be required).

A tentative reference model was developed from the research date that facilitated an ongoing assessment of the sales and marketing capability of the trial.

### The reference model

**Figure 2:**
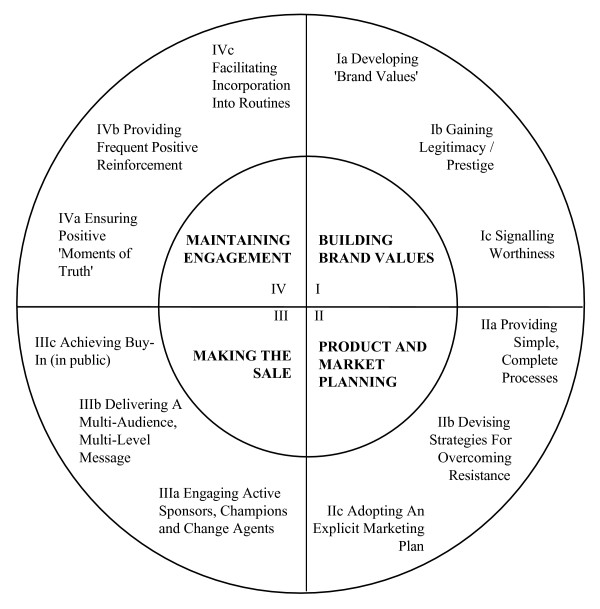
Reference model.

The reference model defines the capabilities required for successful marketing and selling of a medical trial that offered a holistic ideal type that the trial could use to define excellence [17]. It has four domains and 12 components and is illustrated as a wheel diagram (Figure 2). The twelve components are described below.

#### Ia) Developing brand values

Brand values define what a brand is and what it is not – i.e. its personality. A clinical trial can be seen as a brand. Without explicit brand values it is impossible to communicate a coherent and persuasive perception of a trial's promise – i.e. what the trial intends to deliver to medicine, doctors, patients etc.

#### Ib) Gaining legitimacy and prestige

Trials need legitimacy – they need to be positively tagged by association with prestigious individuals and institutions (so a hospital doctor may say, "I know that this is an important trial because Professor X, who I know and respect, is supporting it). Legitimacy and prestige provide persuasive credibility key to gaining access to decision-makers who decide whether a trial should be supported and maintain engagement.

#### Ic) Signalling worthiness

It is vital to signal to likely participants that, "this trial will create greater value than the costs (time, effort or money) involved". Buy-in is more likely to occur when participants realise, and identify with, the potential benefits that will be delivered by the success of the trial. Methods for doing this include presentations at conferences, journal publications, advertising, public relations and training materials.

#### IIa) Providing simple, complete processes

Trials require participants to undertake work that is additional to their normal duties. Providing simple, complete processes reduces the costs of participation and increases the chances that involvement will be affordable.

#### IIb) Devising strategies for overcoming resistance

Potential participants frequently raise objections. Trials should have standard and persuasive answers to these. Having a persuasive answer for each objection increases the probability of making a sale.

#### IIc) Adopting an explicit marketing plan

The marketing of a trial is too important and too complicated to be done informally. A formal marketing plan is required that should include a definition of target market segments (groups that need to buy in to the trial) and the trial's unique selling points (USPs). It is to be expected that the marketing plan will need to be revised frequently – probably every quarter. It can be useful to have separate plans for dealing with (1) *The Uninformed *(Inform and persuade with targeted stories), (2) *The Unconvinced *(Address concerns point-by-point – "get to yes"), (3) *The Laggards *(Enrol, cajole, facilitate and target), (4) *The Steady Performers *(Reward, renew, upgrade and recognise) and (5) *The Stars *(Honour, learn from, and nourish).

#### IIIa) Engaging active sponsors, champions and change agents

Selling a trial to prospective participants requires persuasion. This requires enrolling sponsors (public advocates), champions (activists) and change agents (facilitators). Trial managers need a network of supporters to spread the message. Persuasion is more likely to occur if the advocate is respected and known personally to the prospective participant.

#### IIIb) Delivering a multi-audience, multi-level message

Trials need to convey sales messages through publicity, presentations, training materials, etc. These should be tuned to the distinctive needs of target groups – for example, surgeons are likely to be persuaded by different messages than administrators or nursing staff. Speaking in the language of the person being targeted and addressing their particular pattern of motivation is more likely to succeed than a one size fits all approach.

#### IIIc) Achieving buy-in (in public)

Public buy-in requires that intended participants announce their commitment to join the trial in a setting where others hear them. This is important because when someone states, in public, that they are willing to undertake an action, then they are more likely to abide by their commitment than if they take a silent decision – that can be forgotten easily.

#### IVa) Ensuring positive moments of truth

People evaluate organisations (including trial management teams) on the basis of their experiences at moments of truth. For example, if a doctor has a technical question about entering a patient into a trial she will gain a strong impression of the trial management team's competence by the way that the query is handled. If trialists behave well in a moment of truth then loyalty grows; if not, loyalty diminishes.

#### IVb) Providing frequent positive reinforcement

Positive reinforcement for existing participants should be an important part of a trial's participant retention strategy. It is more expensive to recruit new participants than to retain existing participants.

#### IVc) Facilitating incorporation into routines

Activities that become embedded as routines are more likely to be done than one-offs. Trial procedures should be incorporated into the routines of units undertaking the work.

## Discussion

We found that the CRASH trial faced challenges in marketing and selling that were mission critical – i.e. if goals were not achieved then the trial would fail. Farrell, amongst others, has been arguing for a greater recognition of the role of management in the conduct of clinical trials [5]. The key strength of the study reported here is that, for the first time in academic literature, it offers a reference model that provides a conceptual framework that can support and guide trial managers in assessing their marketing strengths and weaknesses [18]

The reference model described above should be seen as a tentative framework rather than a definitive template. It was developed from a theory-building process from a single trial and is best considered as a set of provocative hypotheses – later they may be developed as provisional audit tools. It may be that the reference model could be used as a diagnostic tool to identify if, and at what points, a trial is failing so that remedial interventions could be undertaken. An audit of the CRASH trial enabled components that were considered to be weaker than others to be identified and initiatives undertaken to improve in these areas. Further research is needed in other trials to explore whether the model is complete and correct and whether useful audit tools can be developed.

Clinical trials are not only research activities- they are also time-bound businesses that have two interdependent sets of processes – one clinical and the other managerial. In the main, since trials are seen as clinical endeavours, they are dominated by clinical issues and led by people with clinical skills. This is essential for certain policies and practices but this cultural bias can result in the managerial aspects of trials being, relatively, neglected. If this is true, even if only in part, it means that the radical improvement of clinical trials could require different ways of defining the challenges of running successful trials – in particular, to ensure that they are seen as management challenges that can benefit from the informed use of selected management processes and techniques.

These considerations suggest that looking within past trials for the answers to the problem of under-performing trials is necessary but will not be sufficient. In order to improve trials it will be necessary to look outside the world of clinical practice, into the worlds of business strategy, management, marketing and sales to gain a fuller understanding of what can be done to upgrade performance of clinical trials. This insight is not new. Donovan, Mills et al [19] state that the, "methodological literature (on trials) is almost exclusively statistical and epidemiological, and very little of it is concerned with the conduct or the particular demands that trials put on trialists and participants" (pp766).

## Conclusion

This study could begin to change the ways that trial managers undertake their work. Also, it provides a different way to think about the skill sets and competencies needed by those who manage clinical trials. In essence, the message of this study is simple – even simplistic. It is that trials are both complex projects and businesses (they need to find customers). The key implication for clinicians is that insufficient attention to management issues and marketing or sales activities will degrade the performance of the trial.

There are significant implications for policy makers and funding bodies as well. If the tentative conclusions of this study are correct, then the funders will need to examine more than the scientific case before sponsoring a trial. They will need to see a marketing and sales plan, and be assured that all of the required elements of the business system will be developed. Since a successful trial requires both good science and good management, both need to be given their due weight.

But there are differences. Business is about profit. Medicine is driven by human values. It would be wrong to infer that publicly funded trials need to be more like businesses – rather, we suggest, that trials may benefit from using business concepts and business techniques.

This cross-disciplinary study was based on the premise that something new would be gained if a researcher from the world of business and management studied a clinical trial from his disciplinary perspective and worked with trialists to devise a useful framework. Since innovation is frequently facilitated by a clash of disciplines, it may be some of the insights needed to improve trial recruitment will come from fields other than medicine.

## Competing interests

The author(s) declare that they have no competing interests.

## Authors' contributions

The idea for STEPS was jointly conceived by the Principal Investigator, MKC, with AMG, VAE, DE, JG, CS, IR, DF, AMM and RCK. DF and IR proposed the case study. All authors contributed to the study design. DF, IR, DE and HS wrote the first draft of the manuscript, and all the authors read and approved the final manuscript. MKC is guarantor for STEPS, and DF is the guarantor for the case study
